# Variant of SNP rs1317082 at CCSlnc362 (RP11-362K14.5) creates a binding site for miR-4658 and diminishes the susceptibility to CRC

**DOI:** 10.1038/s41419-018-1222-5

**Published:** 2018-12-05

**Authors:** Chaoqin Shen, Tingting Yan, Zhenhua Wang, Heng-chuan Su, Xiaoqiang Zhu, Xianglong Tian, Jing-Yuan Fang, Haoyan Chen, Jie Hong

**Affiliations:** 1 State Key Laboratory for Oncogenes and Related Genes, Key Laboratory of Gastroenterology & Hepatology, Ministry of Health, Division of Gastroenterology and Hepatology, Renji Hospital, School of Medicine, Shanghai Jiao Tong University, Shanghai Cancer Institute, Shanghai Institute of Digestive Disease, 145 Middle Shandong Road, 200001 Shanghai, China; 2 Department of Urology, Fudan University Shanghai Cancer Center, Shanghai, China; 3 Department of Oncology, Shanghai Medical College, Fudan University, Shanghai, China

## Abstract

Genome-wide association studies (GWAS) have identified several loci harboring variants that affected the risk of colorectal cancer; however, the specific mechanisms by which germline variation influenced the tumorigenesis of colorectal cancer (CRC) remains unrevealed. We found the T>C variant of rs1317082, locating at the exon 1 of lncRNA RP11-362K14.5 (CCSlnc362), was predicted to be a protective locus for cancer. However, the specific role of CCSlnc362 and the interaction between CCSlnc362 and rs1317082 variation in colorectal cancer and its mechanisms remain unclear. Here we explored the expression and function of CCSlnc362 in CRC cells and tissues. We found lncRNA CCSlnc362 expression was significantly increased in CRC samples. Follow-up functional experiments elucidated that downregulation of CCSlnc362 inhibited cell proliferation, arrested cell cycle, and promoted apoptosis in CRC cells. The T>C variant of rs1317082 at CCSlnc362 exon 1 created a binding site for miR-4658 to reduce the expression of CCSlnc362 and thus decreased the susceptibility to CRC. Our findings have provided supporting evidence for the protective role of rs1317082 variation and the potential oncogenic role of lncRNA CCSlnc362 in CRC. The data shed new light on the relationship between germline variation, miRNAs, and lncRNAs and opened a new avenue for targeted therapy in CRC.

## Introduction

Colorectal cancer (CRC) is one of the most devastating malignancies in the digestive system^1,2^ and is third commonly diagnosed cancer as well as the second fatal cancer^3,4^. Despite of increased uptake of screening, there are still a huge number of new cases of CRC diagnosed at advanced stage^5^. Lack of early specific symptoms and molecular biomarkers, distant metastasis, chemotherapy resistance, and tumor recurrence cooperatively contribute to poor prognosis of CRC. Therefore, it is of great urgency to explore and develop more efficient biomarkers and targets to facilitate the diagnosis and treatment of CRC.

CRC is a multifactorial disease that occurs due to genetic and environmental factors. Over the past decade, >60 loci have been discovered associated with effects on CRC. Yet there remains a great opportunity to discover additional novel genetic regions. A majority of functional single-nucleotide polymorphisms (SNPs) consist of intronic SNPs and exonic SNPs. Characterizing the biological function of these SNPs can provide us with an opportunity to illuminate the progression of CRC and to explore more effective and efficient ways of therapy. In recent years, studies have mostly focused on intron SNPs, whereas exonic SNPs have attracted less attention, especially in CRC, which encourages us to place emphasis on exonic SNPs.

It is estimated that >93% susceptibility loci are located in noncoding regions^6^. Recently, studies revealed that genetic variation of SNP can influence the susceptibility to disease by changing the expression of long noncoding RNAs (lncRNAs)^7,8^. lncRNAs are noncoding transcripts with >200 nucleotides in length^9–11^. LncRNAs have been identified as vital components of risks to develop cancer. Reports showed that lncRNAs, like HOTAIR, FAL1, and GClnc1, promoted carcinogenesis and predicted poor prognosis of patients^12–14^. On the other hand, several studies reported that lncRNAs played protective roles in the development of cancer^15,16^. LINC00673 prohibited SRC–extracellular signal–regulated kinase pathway and activated signal transducer and activator of transcription factor 1-dependent antitumor signaling through ubiquitination of PTENP11^17^. LincRNA-p21 blocked the β-catenin signaling pathway and attenuated the self-renewal of stem cells in CRC^18^. However, a large number of lncRNAs still remain functionally uncharacterized in CRC and their mechanisms need to be further explored.

Combined with the previous investigation, we hypothesized whether exonic SNPs could influence the susceptibility to CRC through lncRNA. By analyzing the location of CRC-associated genetic variants, we found two SNPs locating in exons of lncRNA: rs6983267 (exon of CCAT2) and rs1317082 (exon of RP11-362K14.5). The function of rs6983267 and CCAT2 have been already illustrated in CRC^19,20^. However, the relationship between rs1317082 variant and RP11-362K14.5 expression have never been reported before. In the current study, we confirmed that T>C variant of rs1317082 played a protective role in CRC. Mechanistically, rs1317082 may create binding sites for miR-4658 to reduce the expression of lncRNA RP11-362K14.5. RP11-362K14.5 may function as an oncogenic lncRNA to initiate CRC by promoting cell proliferation and decreasing cell apoptosis. Therefore, we designated RP11-362K14.5 as a CRC SNP-associated lncRNA RP11-362K14.5 (CCSlnc362).

## Materials and methods

### Clinical specimen collection

CRC tissues and adjacent non-tumor tissues were collected from patients who underwent surgery at Renji Hospital Affiliated to Shanghai Jiao Tong University School of Medicine from January 2011 to December 2013 (Renji Cohort 1). Tissues were preserved at −80 °C immediately after removal from patients in case of degradation. None of the patients enrolled had received chemotherapy, hormone therapy, or radiotherapy before surgery. The study was approved by the Ethics Committee of Renji Hospital Affiliated to Shanghai Jiao Tong University School of Medicine. All the patients participated in the study had signed informed consent before enrollment.

### Bioinformatics analysis

The genotypes of colorectal tissues and CRC cells were analyzed in Sangon Biotech (China). The free energy of CCSlnc362 was calculated by RNAhybrid (https://bibiserv.cebitec.uni-bielefeld.de/rnahybrid/). The binding sites of CCSlnc362 and mir-4658 and local folding structures were predicted using RegRNA 2.0 (http://regrna2.mbc.nctu.edu.tw). The expression quantitative trait locus (eQTL) data were analyzed using GTEx portal (https://www.gtexportal.org/home/).

### Cell cultures

The immortalized human colorectal epithelial cell line (FHC) and CRC cell lines HCT116, DLD-1, SW480, LOVO, HT29, and RKO were purchased from American Type Culture Collection (ATCC). SW1116 and SW620 cells were obtained from the Cell Resource Center, Shanghai Institute of Biochemistry and Cell Biology at the Chinese Academy of Sciences. All cells were cultivated in a humidified environment with 5% CO_2_ at 37 °C in recommended growth medium added with 10% fetal bovine serum. FHC cells: Dulbecco’s modified Eagle’s medium/F12 Medium (Gibco, USA); SW1116, SW620 and DLD-1 cells: RPMI-1640 Medium (Gibco, USA); HCT116 and HT29 cells: McCoy’s 5a Medium (Gibco, USA); SW480 cells: L-15 medium (Gibco, USA); LoVo cells: F-12K Medium (Gibco, USA).

### Cell treatments

Three types of small interfering RNAs (siRNAs) against CCSlnc362 were synthesized at GenePharma Technologies (Shanghai, China) and transfected into CRC cell lines with DharmaFECT 1 siRNA transfection reagent (Thermo Scientific, USA). The transfection efficiency was examined by quantitative real-time PCR (qRT-PCR). CCSlnc362-siRNA with the highest knockdown efficiency was chosen for further study. Nonspecific siRNA was used as a negative control. The sequences targeting CCSlnc362 were listed as follows: 5’-GCUAGAAUCAACAUACUUATT-3’; 5’-CUCGAGUUCUGAAUUAUCATT-3’; 5’-CGUGAUGUGAGAGUUACAATT-3’.

### Total RNA extraction and qRT-PCR

Total RNA of CRC tissues, adjacent non-tumor tissues, and CRC cell lines were extracted using trizol reagent (Takara, Japan). The first-strand cDNA was synthesized using 1 μg total RNA by a PrimeScript RT Reagent Kit (Takara, Japan) for lncRNA analysis. For miRNA analysis, the first-strand cDNA was synthesized using 500 ng total RNA by the Mir-X^TM^ miRNA First-Strand Synthesis Kit (Takara, Japan). PCR was performed using ABI reagent (Thermo Fisher Scientific, USA) in the StepOnePlus RT-PCR system (Applied Biosystems, USA). All the operations were conducted according to the manufacturer’s guidance.

2^−ΔΔCt^ method was used to quantify the relative expression levels^21,22^. Glyceraldehyde 3-phosphate dehydrogenase (GAPDH) and U6 were used as internal controls. The primers used in this study were listed as follows:

CCSlnc362-Forward: TTTGGCTGTGATTTTCCACGTT;

CCSlnc362-Reverse: TGCAAATAAACTGCCCACACCT;

GAPDH-Forward: GCATTGCCCTCAACGACCAC;

GAPDH-Reverse: CCACCACCCTGTTGCTGTAG.

### Cell proliferation analysis

CRC cell lines HCT116 and DLD-1 cells were seeded into 96-well plates at a density of 2000 cells per well. CCSlnc362-siRNA and control-siRNA were transfected accordingly into cells using DharmaFECT 1 siRNA transfection reagent the next day. Cell Counting Kit-8 (Dojindo Molecular Technologies, Japan) was used to incubate with cells away from light for 2 h at 37 °C at the time points of 24, 48, 72, 96, and 120 h after plantation. The absorbance was measured with a wavelength 450 nm (450 OD). The cell proliferation curves were plotted using the absorbance detected at each time point. The experiments were repeated for three times and each group was conducted in six replicate wells.

### Cell cycle analysis by flow cytometry

CRC cell lines HCT116 and DLD-1 cells were seeded into 6-well plates at a density of 3 × 10^5^ cells per well. CCSlnc362-siRNA was transfected into cells using DharmaFECT 1 siRNA transfection with nonspecific siRNA as a negative control. The cells were collected 48 h after transfection, centrifuged at 4 °C for 5 min and washed with ice-cold phosphate-buffered saline (PBS). The cells were then fixed with 75% ethanol at −20 °C overnight. The cells were centrifuged at 4 °C for 5 min the next day and the supernatant was removed. After two times of washing with PBS, the cells were treated with 10 μL RNase and incubated at 37 °C for half an hour. At last, 50 μL propidium iodide (PI) was added into each tube and incubated with cells for 15 min in the dark. The fluorescence-activated cell sorting (FACS) flow cytometer (BD Biosciences, San Jose, CA, USA) was used for cell cycle analysis.

### Cell apoptosis analysis by flow cytometry

CRC cell lines HCT116 and DLD-1 cells were seeded into six-well plates and CCSlnc362-siRNA was transfected into cells the next day. The cells and the supernatant were all harvested into centrifuge tubes 48 h later. The apoptosis analysis was performed using Annexin V-FITC Apoptosis Detection Kit (Invitrogen, USA). Cells were washed with binding buffer, incubated with Annexin V-fluorescein isothiocyanate (FITC) in the dark for 30 min and then stained with PI for 5 min away from light. Finally, the FACS flow cytometer was used for cell apoptosis analysis.

### Luciferase reporter assays

The reporter plasmids bearing a CCSlnc362 fragment with the rs1317082 [T] (CCSlnc362 [T]) or rs1317082 [C] allele (CCSlnc362 [C]) were constructed at Generay Technologies (China). The miR-4658 mimics and miR-4658 inhibitors were designed and synthesized at GenePharma Technologies (China). HCT116 cells were planted into 96-well plates at a density of 4000 cells per well. Reporter plasmids were cotransfected with miR-4658 mimics (or miR-4658 inhibitor) and Renilla vector using Lipofectamine 3000 (Invitrogen, USA). Cells were harvest and the luciferase activities were detected using the Dual-Luciferase Reporter Assay Kit (Promega, USA). Firefly luciferase activity and Renilla luciferase activity were measured using FLUOstar Omega (BMG LABTECH, German). The results were showed in the form of relative firefly luciferase activity normalized to Renilla luciferase activity. All operations above were performed according to the manufacturer’s instruction. The sequences of miR-4658 mimics and inhibitors involved in the study are listed as follows: miR-4658 mimics: GUGAGUGUGGAUCCUGGAGGAAU; miR-4658 inhibitor: AUUCCUCCAGGAUCCACACUCAC. All the experiments were repeated for three times and four replicates were conducted for each group.

### Statistical analysis

All the statistical analyses in this study were carried out using the SPSS 19.0 software (SPSS Inc, Chicago, IL, USA) and GraphPad Prism 6 (San Diego, CA, USA). Results were presented as means ± SEM (standard error of mean). Student’s *t* test was used for comparison of means between two groups. *P* < 0.05 was considered to be significant difference.

## Results

### T>C variant of rs1317082 plays a protective role in CRC tumorigenesis and impairs the expression of CCSlnc362 in colorectal tissues and CRC cells

**Fig. 1:**
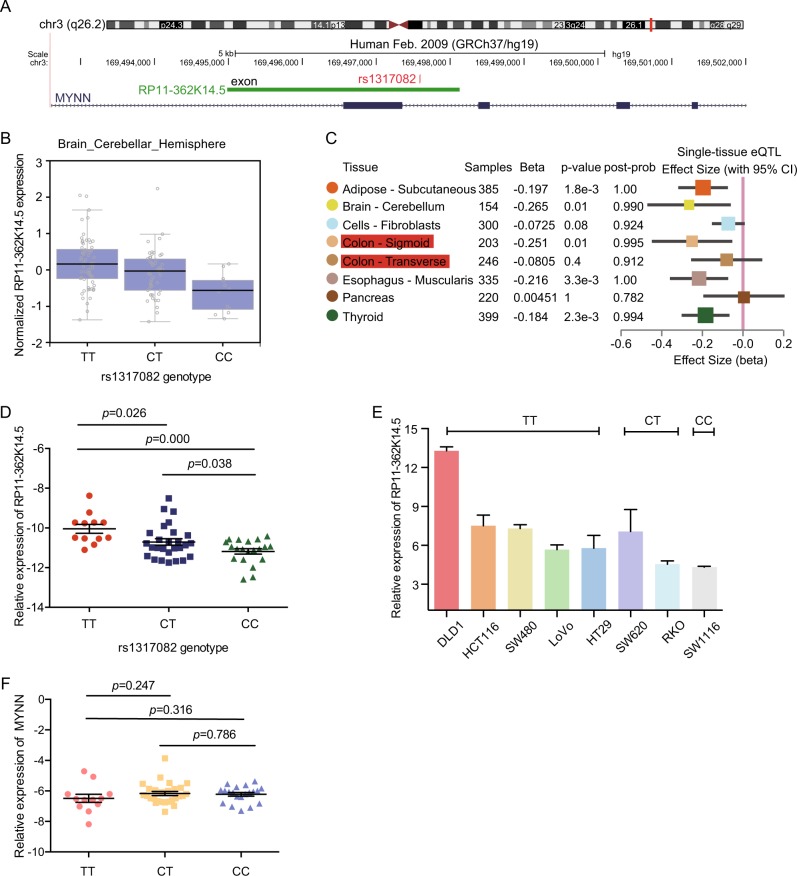
The association between rs1317082 variant and RP11-362K14.5 expression. **a** Rs1317082 was located in the exon of RP11-362K14.5 in UCSC. **b** Rs1317082 eQTL analysis of CCSlnc362 expression in brain and cerebellar hemisphere tissues. **c** RP11-362K14.5 and rs1317082 eQTL in multiple tissues. **d** RP11-362K14.5 expression was determined by real-time PCR in normal colorectal tissues in subjects with TT (*n* = 12), CT (*n* = 27), and CC (*n* = 20) genotypes at rs1317082. Results are normalized to GAPDH. **e** The relative levels of RP11-362K14.5 was measured in eight CRC cell lines with different rs1317082 genotype. **f** The relative expression of MYNN was measured by real-time PCR in normal colorectal tissues in subjects with TT (*n* = 12), CT (*n* = 27), and CC (*n* = 20) genotypes at rs1317082. Results are normalized to GAPDH

Genome-wide association studies (GWAS) have identified >60 loci harboring variants that affected the risk of colorectal tumorigenesis (Supplementary Figure 1). Even though exonic SNPs are important parts of functional SNPs^23^, there are still a large number of exonic SNPs that remain functionally uncharacterized in CRC and their mechanisms need to be further explored. Through analyzing the CRC-associated susceptibility loci in UCSC, we found that rs1317082 was located in exon 1 of RP11-362K14.5 (Fig. 1a). Nevertheless, little was known about the potential involvement of rs1317082 in CRC. Meta-analysis of three GWAS identified rs10936599, the lead SNP of rs1317082, as a protective locus for CRC (odds ratio (OR) = 0.93, *P* = 3.39 × 10^−8^)^24^. Hence we focused on the potential role of rs1317082 in CRC tumorigenesis and the specific mechanism.

To date, quantities of studies have revealed that SNPs located in lncRNA may influence the expression level of lncRNA^17^. We hypothesized that the expression of RP11-362K14.5 was influenced by genotypes of rs1317082. As we have known, GTEx (genotype-tissue expression) project has comprehensively analyzed the relationship between genetic variation and the expression of transcripts in various human tissues to determine how genomic variation affects the gene expression^25^. First of all, we sought eQTL data in GTEx Portal. The eQTL analysis revealed that the relative expression of RP11-362K14.5 in the brain and cerebellar hemisphere tissues was affected by rs1317082 genotype (Fig. 1b). In addition, we further hunted for single-tissue eQTL information in GTEx Analysis Release V7 (dbGaP Accession phs000424.v7.p2). The data showed rs1317082 variant decreased the level of RP11-362K14.5 in colon tissues (Fig. 1c). In order to validate the above consequence in GTEx, we obtained normal colorectal tissues, detected the relative expression of RP11-362K14.5, and analyzed the genotype of rs1317082 for each sample. As expected, the patients whose genotype was CC at rs1317082 had significantly lower expression of RP11-362K14.5 compared with those patients whose genotype was CT or TT at rs1317082 locus (Fig. 1d), which was consistent with the previous speculation. Furthermore, we collected eight CRC cell lines (DLD-1, HCT116, SW480, LoVo, HT29, SW620, RKO, and SW1116) and identified the genotypes of these cells. The levels of RP11-362K14.5 were quite different in these eight CRC cells with different rs1317082 genotype. As shown in Fig. 1e, SW1116 cells with CC genotype at rs1317082 had the lowest expression of RP11-362K14.5, while other cells with CT/TT genotype at rs1317082 had relatively higher expression levels of RP11-362K14.5. As Fig. 1a shows, rs1317082 was located at the exon of RP11-362K14.5 and located in the intron of MYNN (myoneyrin) as well. To examine whether rs1317082 locus influenced the level of MYNN, we detected the relative expression of MYNN in patients with different rs1317082 genotype. RT-PCR data showed that there was no significant difference of MYNN expression in CRC patients with different rs1317082 genotype (Fig. 1f). The data suggested that T>C variant of rs1317082 decreased the expression levels of RP11-362K14.5 in colorectal tissues and CRC cells. Therefore, we designated RP11-362K14.5 as a CRC SNP-associated lncRNA RP11-362K14.5 (CCSlnc362).

### miR-4658 targets CCSlnc362 containing rs1317082 [C] allele in CRC

**Fig. 2:**
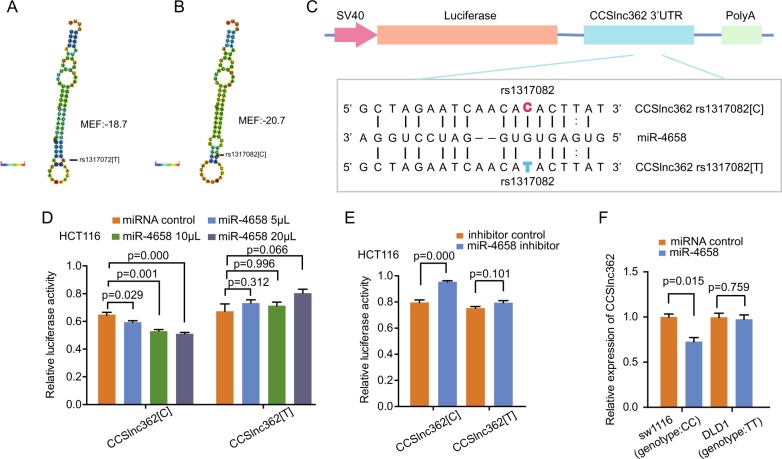
miR-4658 targets CCSlnc362 containing rs1317082 [C] allele in CRC. **a**, **b** Predicted folding structures and minimum free energy (MFE) for CCSlnc362 with rs1317082 [T] or rs1317082 [C]. We used RegRNA 2.0 and RNAhybird. **c** Predicted interaction between miR-4658 and CCSlnc362. **d**, **e** Relative luciferase activities of CCSlnc362 fragment with the rs1317082 [T] or rs1317082 [C] cotransfected with miR-4658 mimics or miR-4658 inhibitor were calculated in HCT116 cells. **f** Relative expression of CCSlnc362 was detected in SW1116 (genotype: CC) and DLD-1 cells (genotype: TT) transfected with miR-4658 mimics. All the experiments were repeated for at least three times and four replicates were conducted for each group

Previous studies revealed that SNPs located in lncRNA could create or decrease binding sites for specific miRNA and thus influence the expression levels of lncRNA^17^. And it is widely acknowledged that miRNA can interact with lncRNA and regulate its expression^26–28^. Therefore, we hypothesized the T>C variant of SNP rs1317082 in CCSlnc362 could affect CCSlnc362 expression by altering a specific microRNA-binding sites. As expected, the free energy of CCSlnc362 as well as its local folding structures were changed in the situation of T>C variant at rs1317082 (Fig. 2a, b). Bioinformatics analysis revealed that T>C variant of rs1317082 could increase the binding efficiency of miR-4658 in CCSlnc362 (Fig. 2c). We next constructed the luciferase reporter plasmids bearing CCSlnc362 fragment with the rs1317082 [T] (CCSlnc362 [T]) or rs1317082 [C] allele (CCSlnc362 [C]). The recombination reporter plasmids and miR-4658 mimics were transfected to HCT116 cells. Luciferase assay data showed that miR-4658 mimics significantly reduced the luciferase activities of the construct containing rs1317082 [C] allele in a dose-dependent manner but not in the construct containing rs1317082 [T] allele (Fig. 2d). We then cotransfected the reporter plasmids to HCT116 cells with miR-4658 inhibitor and found that the luciferase activity was significantly enhanced in cell transfection with reporter plasmid containing CCSlnc362 fragment with rs1317082 [C]. Likewise, there was no significant change of luciferase activity in cell transfection with reporter plasmid containing CCSlnc362 fragment with rs1317082 [T] (Fig. 2e). To confirm whether the interaction between CCSlnc362 and miR-4658 existed in an allele-specific manner, we transfected SW1116 cells (genotype: CC) with miR-4658 mimics. RT-PCR data showed that CCSlnc362 expression was remarkably decreased in SW1116 cells with miR-4658 mimic transfection. However, the phenomenon was not observed in DLD-1 cells (genotype: TT) with miR-4658 mimic transfection (Fig. 2f), indicating that miR-4658 may target CCSlnc362 containing rs1317082 [C] allele (CCSlnc362 [C]) but not CCSlnc362 containing rs1317082 [T] allele (CCSlnc362 [T]).

**Fig. 3:**
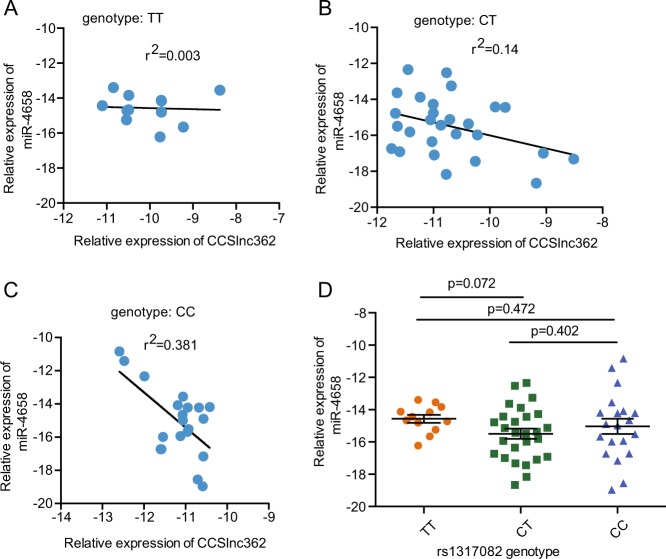
The association between CCSlnc362 and miR-4658. **a**–**c** The association between relative expression of CCSlnc362 and miR-4658 in normal colorectal tissues with different genotype groups of rs1317082. **d** Relative expression of miR-4658 in colorectal tissues with different genotype groups of rs1317082. Results are normalized to GAPDH or U6

We next explore the correlation between CCSlnc362 and miR-4658 in CRC patients with different genotype of rs1317082 locus. RT-PCR assay revealed that CCSlnc362 levels were negatively related with miR-4658 in CRC patients with CC and CT genotype at rs1317082, whereas there was no correlation between CCSlnc362 and miR-4658 expression in CRC patients with TT genotype at rs1317082 (Fig. 3a–c). We further assessed the influence of rs1317082 genotype on the expression of miR-4658 in these CRC patients. However, the correlation is not significant between miR-4658 levels and the genotypes at rs1317082 (Fig. 3d). The combined analysis suggested that miR-4658 could interact with CCSlnc362 containing rs1317082 [C] allele (CCSlnc362 [C]) and decrease lncRNA CCSlnc362 expression in CRC.

### The expression of CCSlnc362 is significantly increased in CRC cell lines and tissues

**Fig. 4:**
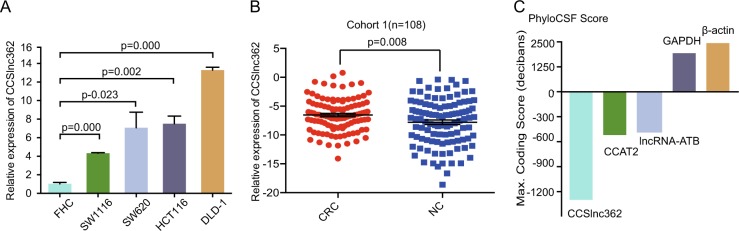
The expression of CCSlnc362 in CRC cell lines and tissues. **a** The expression of CCSlnc362 was measured by real-time PCR in FHC and CRC cell lines with different rs1317082 genotype. Results are normalized to GAPDH and shown as means ± SEM. *P* values are from two-sided Student’s *t* tests. **b** The relative expression of CCSlnc362 was detected in 108 pairs of colorectal cancer tissues and adjacent normal tissues. Results are normalized to GAPDH and shown as means ± SEM. **c** The coding potential was analyzed for the sequences of CCSlnc362, CCAT2, lncRNA-ATB, GAPDH, and β-actin across 29 mammals in all three reading frames using PhyloCSF. PhyloCSF scores >0 were considered to have coding potential, whereas scores <0 suggested no coding potential

Based on our previous study, we speculated that CCSlnc362 was an oncogenic factor in CRC. To verify our hypothesis, we measured the expression of CCSlnc362 in an immortalized human colorectal epithelial cell line (FHC) and CRC cell lines (SW1116, SW620, HCT116, and DLD-1). RT-PCR data showed that CCSlnc362 expression was increased in CRC cell lines, compared with normal colorectal epithelial cell line (Fig. 4a). To verify the association between CCSlnc362 expression and CRC progression, we examined its abundance in 108 pairs of fresh CRC tissues and adjacent non-tumor colorectal tissues. RT-PCR data showed that the expression of CCSlnc362 was much higher in CRC tissues than those of non-tumor colorectal tissues (Fig. 4b). In order to determine whether CCSlnc362 was a novel lncRNA, the coding potential was analyzed for the CCSlnc362 sequence across 29 mammals in all three reading frames using PhyloCSF^29^. LncRNA-ATB and CCAT2 served as control noncoding gene and GAPDH and β-actin acted as control coding gene in the analysis. CCSlnc362 had a low PhyloCSF score compared with GAPDH and β-actin, indicating that CCSlnc362 almost had no protein-coding potential (Fig. 4c). These data suggested that CCSlnc362 was a novel lncRNA and upregulated in CRC tissues.

### CCSlnc362 promotes cell proliferation and cell cycle in CRC cell lines

**Fig. 5:**
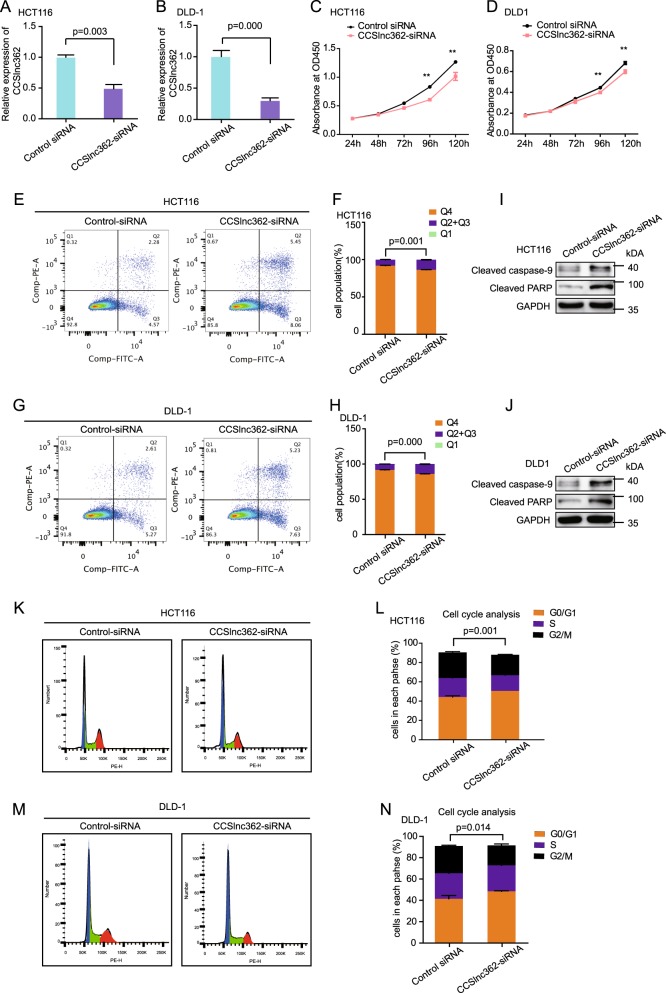
Down-regulation of CCSlnc362 leads to inhibition of proliferation, cell cycle arrest, and promotion of apoptosis. **a**, **b** Knockdown efficiency was confirmed by real-time PCR in HCT116 cells and DLD-1 cells. Results are normalized to GAPDH. **c**, **d** Cell proliferation was estimated by using the CCK-8 in HCT116 and DLD-1 cells transfected with CCSlnc362-siRNA. The experiment was performed in six replicate wells for each group. **e**- **h** Cell apoptosis analysis was conducted in HCT116 and DLD-1 cells transfected with CCSlnc362-siRNA using flow cytometry. **i**, **j** Western blot was used to detect the expression of cleaved caspase-9 and cleaved PARP in HCT116 and DLD-1 cells transfected with CCSlnc362-siRNA. **k**–**n** Cell cycle analysis was performed in HCT116 and DLD-1 cells transfected with CCSlnc362-siRNA using flow cytometry. ** indicated p<0.01

To elucidate whether CCSlnc362 played a role in tumorigenesis of CRC, we transfected CCSlnc362-siRNA into CRC cell lines DLD-1 and HCT116 (with TT genotype at rs1317082). CCSlnc362-siRNA significantly reduced CCSlnc362 RNA level in HCT116 (Fig. 5a) and DLD1 cells (Fig. 5b). Knockdown of CCSlnc362 dramatically impaired cell proliferation in HCT116 (Fig. 5c**)** and DLD-1 cells (Fig. 5d). To determine the mechanism by which downregulation of CCSlnc362 inhibited cell growth, we evaluated the oncogenic properties and effects of CCSlnc362 on cell apoptosis by flow cytometry and western blot. Flow cytometric data revealed that transfection of CCSlnc362-siRNA significantly increased cell apoptosis both in HCT116 (Fig. 5e, f) and DLD-1 cells (Fig. 5g, h). Western blot data showed that cleaved caspase-9 and cleaved poly ADP-ribose polymerase, which have been regarded as the marker for apoptosis, were significantly increased in HCT116 (Fig. 5i) and DLD-1 cells (Fig. 5j) with CCSlnc362 downregulation. In addition, knockdown of CCSlnc362 led to an increased cell population in G0/G1 phase and a concomitantly decreased cell population in G2/M phase in HCT116 cells (Fig. 5k, l). Consistently, the percentage of cells in G0/G1 phase increased from 41.45% to 48.44%, while the percentage of cells in G2/M phase decreased dramatically from 24.8% to 18.8% in DLD cells in response to CCSlnc362-siRNA transfection (Fig. 5m, n). Taken together, these data strongly suggested that CCSlnc362 promoted the tumorigenesis of CRC by promoting cell proliferation, accelerating cell cycle, and suppressing cell apoptosis.

## Discussion

CRC has been one of the most malignant tumors with increasing morbidity and mortality^30,31^, and it is a multifactorial disease that occurs due to genetic and environmental factors. GWAS have documented a powerful statistical relationship between genetic loci and human traits. A risk-associated variant at rs7463708 increased binding of ONECUT2, a transcriptional factor, at a distal enhancer that loops to the lncRNA-PCAT1 promoter and thus promotes cancer cell proliferation and tumor growth^8^. SNP rs6939340 contributed to lncRNA-NBAT-1 differential expression and increased cell proliferation and invasion in neuroblastoma^7^. The rs1317082 locates at the exon of CCSlnc362 (RP11-362K14.5) as assessed on UCSC genome browsers. Previous studies reported that the locus rs1317082 was associated with inter-individual leukocyte telomere length^32–34^. However, there was little research on the association between rs1317082 and CRC. CRC risk variant rs1317082 was tightly in linkage disequilibrium with rs10936599 (*r*^2^ = 1). Meta-analysis of three GWAS identified rs10936599, the lead SNP of rs1317082, as a protective locus for CRC^24^. In our current study, we identified rs1317082 variation T>C as a protective factor for CRC.

Recent years, lncRNAs have attracted much attention since lncRNAs have been identified as vital components of risks to develop cancer^35,36^. Several lncRNAs have been reported to be aberrantly expressed in CRC and participate in the process of tumorigenesis. For instance, CCAL induced multidrug resistance (MDR) in CRC through activating Wnt/β-catenin signaling by suppressing AP-2α and further upregulating MDR1/P-gp expression^37^. LncRNA SPRY4-IT1 could act as a ceRNA of miR-101-3p to promote proliferation and invasion in CRC^38^. In the present study, we first identified lncRNA CCSlnc362 as an oncogenic factor in CRC. The expression of CCSlnc362 was proved to be significantly increased in CRC cell lines and CRC tissues. Biological analysis validated that downregulation of CCSlnc362 dramatically suppressed cell proliferation, arrested cell cycle, and simultaneously accelerated apoptosis in CRC cells. Taken together, these data consistently pointed to the notion that CCSlnc362 might serve as a decisive factor in carcinogenesis of human CRC. Nevertheless, the underlying molecular mechanisms that mediated lncRNA expression remain largely unknown. Typically, functional SNPs could be located in coding regions, but cancer susceptibility loci identified by GWAS overwhelmingly occurred in noncoding regions. Therefore, we paid more attention to the influence of susceptibility loci rs1317082 on CCSlnc362 expression. The mapping of eQTL provides a strong approach to elucidate the genetic components that alter gene expression. We performed eQTL analysis in our study and found CCSlnc362 variant resulting from T>C variant at rs1317082 could result in a decreased expression of CCSlnc362 in colorectal cells as well as colorectal tissues, which validated the eQTL data in GTEx project. These results, along with previous reports, highlight the importance of germline variation in the regulation of oncogenic lncRNAs.

**Fig. 6:**
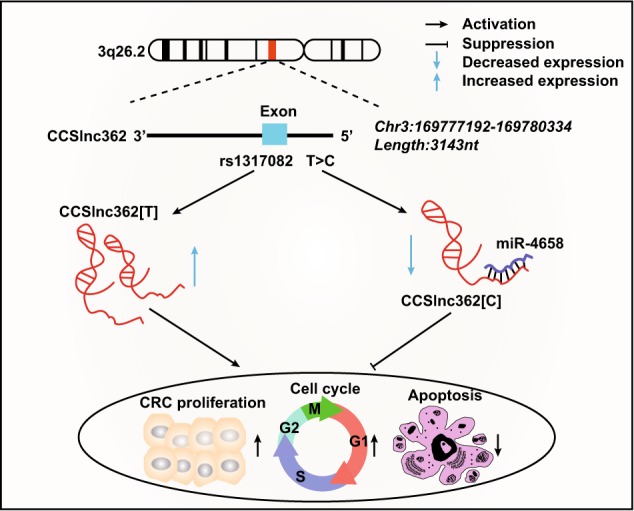
Schematic model of CCSlnc362 functions in colorectal carcinogenesis. CCSlnc362 on chromosome 3q26.2 is tumor-promoting lncRNA in colorectal cancer, which promotes cell proliferation and cell cycle and inhibits apoptosis. Rs1317082 has been reported as a protective factor in tumor and located in exon 1 of CCSlnc362. The T>C variant at rs1317082 creates a binding site for miR-4658 at CCSlnc362 and reduces the expression of CCSlnc362, which eventually diminishes the oncogenic effect of CCSlnc362 in colorectal cancer

It has been shown miRNAs may directly interact with lncRNAs or function in a non-canonical manner to regulate the levels of lncRNA^39–42^. SNPs located in lncRNA could create or decrease binding site for specific miRNA and thus influence the expression levels of lncRNA^43^. Little investigation was reported on the association among germline variation, miRNAs, and lncRNAs in CRC, which encouraged us to explore their interaction in our follow-up study. We found that miR-4658 was involved in the mechanism by which T>C variant at rs1317082 influenced CCSlnc362 expression. The T>C variant at rs1317082 created binding sites at CCSlnc362 for miR-4658 and diminished the tumor-promoting role of CCSlnc362 in CRC and eventually prohibited cell proliferation and cell cycle in CRC (Fig. 6).

Given the significance of CCSlnc362 in both clinical and functional roles in CRC, we regarded CCSlnc362 as a tumor-promoting factor in carcinogenesis and a potential biomarker of CRC. T>C variant of rs1317082 promoted binding efficiency of miR-4658 with CCSlnc362 to reduce this lncRNA expression and eventually decreased the susceptibility to CRC. These data highlighted a vital relationship among germline variation, miRNAs, and lncRNAs and opened a new avenue for targeted therapy for CRC.

## Supplementary information

Supplementary Figure 1

Supplementary figure legends
