# Supplementary figures and images for: Variant of SNP rs1317082 at CCSlnc362 (RP11-362K14.5) creates a binding site for miR-4658 and diminishes the susceptibility to CRC
Source: Cell Death Dis. 2018 Dec 5;9(12):1177. doi: 10.1038/s41419-018-1222-5 (PMC6281592; doi:10.1038/s41419-018-1222-5)

Supplementary Figure 1

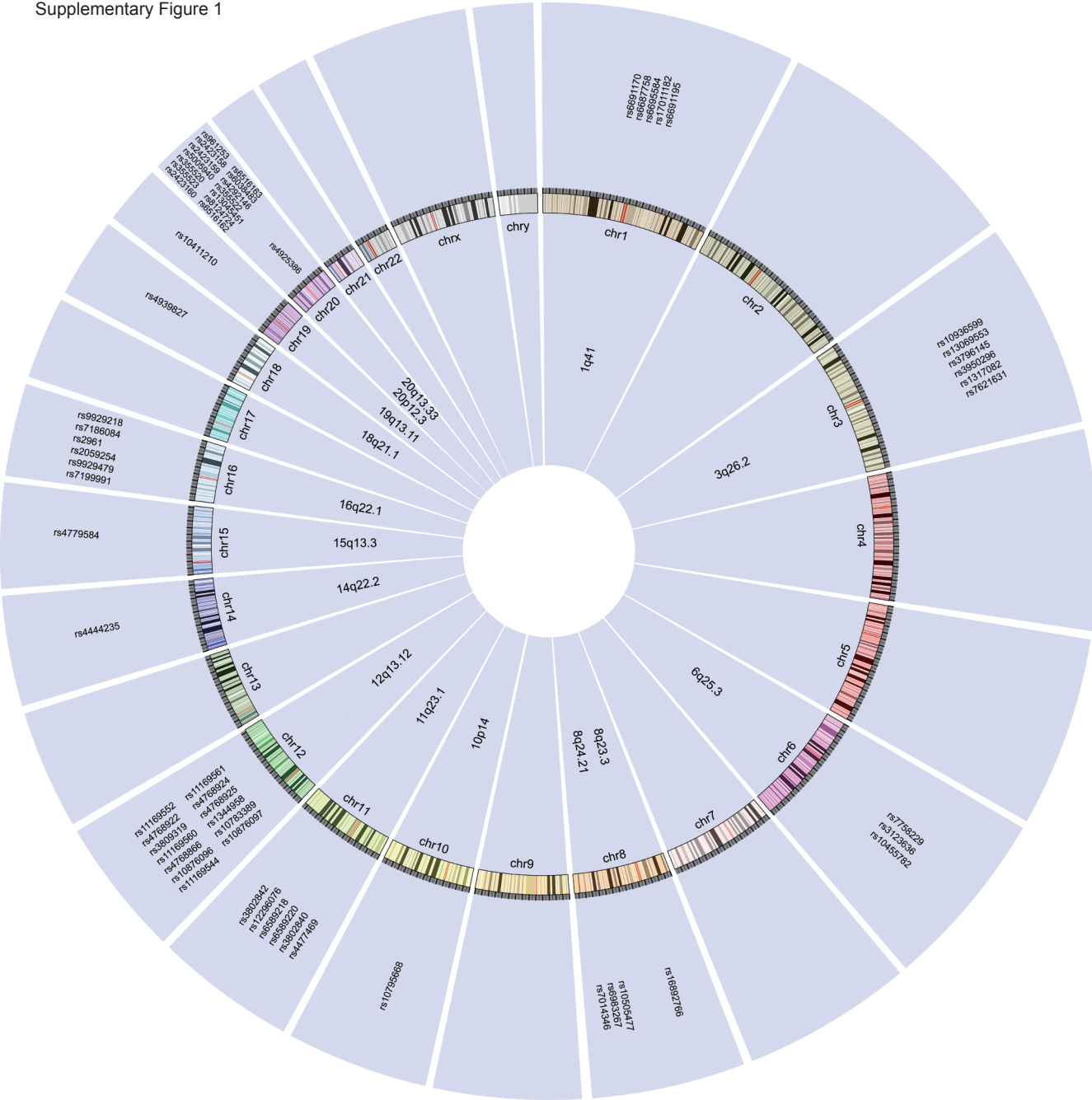

Supplement: Supplementary file 1 — Supplementary Figure 1 [file 41419_2018_1222_MOESM1_ESM.pdf]
