# Supplementary material for: Variant of SNP rs1317082 at CCSlnc362 (RP11-362K14.5) creates a binding site for miR-4658 and diminishes the susceptibility to CRC
Source: Cell Death Dis. 2018 Dec 5;9(12):1177. doi: 10.1038/s41419-018-1222-5 (PMC6281592; doi:10.1038/s41419-018-1222-5)
Supplement: Supplementary file 2 — Supplementary figure legends [file 41419_2018_1222_MOESM2_ESM.docx]

**Supplementary Figure 1: Schematic model of CRC associated SNPs.** Schematic model of 63 loci harboring variants that affected the risk of colorectal tumorigenesis.
